# Mesenteric cystic lymphangioma in adult: a case series and review of the literature

**DOI:** 10.1186/1471-2482-13-S1-A4

**Published:** 2013-09-16

**Authors:** Giovanni Aprea, Francesco Guida, Alfonso Canfora, Antonio Ferronetti, Antonio Giugliano, Melania Battaglini Ciciriello, Antonio Savanelli, Bruno Amato

**Affiliations:** 1 Department of Gastroenterology, Endocrinology and Surgery, General Surgery Division, University “Federico II” of Naples, Via Pansini, 5, 80131, Naples, Italy

## Introduction

Lymphangiomas are rare benign tumors. They are preferentially located in the head, neck, and axilla in children.[1]

However, lymphangiomas in the peritoneal cavity are extremely rare, particularly in adults. In the abdomen, lymphangiomas occur most commonly in the mesentery, followed by the omentum, mesocolon, and retroperitoneum. The etiology is unclear, but they are considered primarily to congenital in origin. [2]

Preoperative diagnosis is often difficult due to the frequent silent clinical course. Radiological investigations are a useful diagnostic tool, but definitive diagnosis is confirmed by histopathology after a complete surgical resection. [3]

The report describes five cases of adult patients with mesenteric lymphangioma.

## Case report N.1

A 67-year-old female presented with a dull abdominal pain in the right hypochondrium and flank of a 1-month duration and presence of constipation.

An abdominal examination revealed distension, hyperactive bowel sounds and tenderness during palpation in the central abdominal quadrant and right flank.

An ultrasonographic abdominal scan revealed the presence of a cystic mass (max diameter 32 mm) in the retrocecal adipose tissue.

A computed tomography (CT) abdomen scan confirmed the presence of the cystic lesion and its dissociability from the cecum, appendix and ileum. In addition, the CT scan revealed the presence of multiple ipodense formations in the body and tail of pancreas.

Neoplastic markers (AFP, CEA, CA 19-9, CA 15-3, CA125, TPS) were negative.

**Figure 1:**
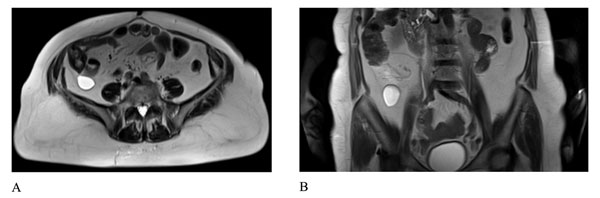
MRI of the abdomen. Axial (a) and coronal (b) T2-weighted TSE images showed the presence of a fluid-filled cystic lesion with a diameter of 35 mm in the retrocecal adipose tissue.

During the hospital stay an abdominal MR was performed. The exam revealed in the retrocecal adipose tissue the presence of a fluid-filled formation with a diameter of 35 mm that appeared indissociable from the cecum for the presence of a tissutal connection (Figure 1 A – B). Other cystic lesions (communicating with pancreatic duct) were present in the whole pancreas and in the right kidney.

The patient underwent exploratory surgery: at laparotomy a yellowish ovalar formation with a diameter of 6 cm was found arising from the mesentery of ileum with partial adhesion of the appendix. The lesion had a cystic aspect and was fluid-filled. We performed a total excision of the lesion and appendicectomy. Histopathological examination was consistent with the diagnosis of mesenteric lymphangioma. The patient has been followed-up for 6 months and no recurrence occurred.

## Case report N.2

A 68-year-old male patient presented with a painless abdominal swelling of two years duration. A clinical examination revealed the presence of an abdominal mass extending from the right hypocondrium to the right iliac fossa.

Abdominal ultrasonography and CT scans revealed the presence of an ipodense and fluid – filled formation, 10 cm in diameter which extended from the right hepatic lobe to the ascending colon. Posteriorly the mass bordered the anterior aspect of the right kidney. In addition, a left kidney agenesia was present.

**Figure 2:**
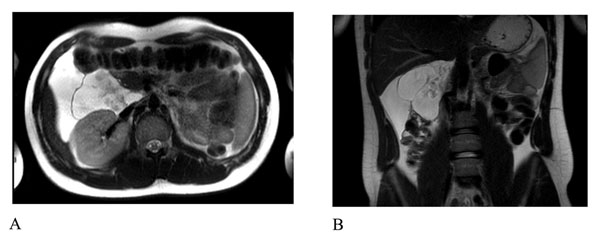
MRI of the abdomen. Axial (a) and coronal (b) T2-weighted TSE images showed the presence of a huge fluid-filled mass with internal septa located in the hepato-renal space extending from the hepatic hilum to the right colon and displacing medially the second duodenal segment and anteriorly the hepatic flexure of the colon.

An abdomen MR was also performed: presence of a 10-cm fuid – filled mass with internal septa that extends from the hepatic hilum to the right colon, displacing medially the second duodenal portion and anteriorly the right colonic flexure. At MR this formation presented a strict contiguity with the common bile duct (Figure 2 A – B).

In consideration of the clinical symptoms, we performed a laparotomic exploration.

At laparotomy we found an ovalar formation of cystic aspect, 10 cm in diameter, which was closely adherent to the biliary tree. The dissection was conducted closely the CBD and the mass appeared originating from the cranial extremity of the common hepatic duct. The complete excision determined a microperforation of the biliary tree that was repaired with a reabsorbable suture. An abdominal drainage was positioned and it was removed in the 7 th postoperative day.

A citologic examination was conducted on the fluid aspirated from the cyst and it revealed the presence of chronic inflammatory cells. The histopathological examination showed the presence of dilated lymphatic vessels and endothelial proliferation: this pattern was consistent with the diagnosis of cystic emolymphangioma.

## Case report N.3

A 80 – year – old male patient complained of diffuse abdominal pain. A clinical examination revealed an occlusive state. We performed an abdominal ultrasonography and CT that revealed in left hypocondrium the presence of an anechogenic ovalar formation of 8,6 x 4 x 5 cm, with internal septa and a thin wall, with polycyclic edge . This formation appeared adherent to the gastric body and the jejunal loops.

Neoplastic markers (AFP, CEA, CA 19-9, CA 15-3, CA125, TPS) were negative.

**Figure 3:**
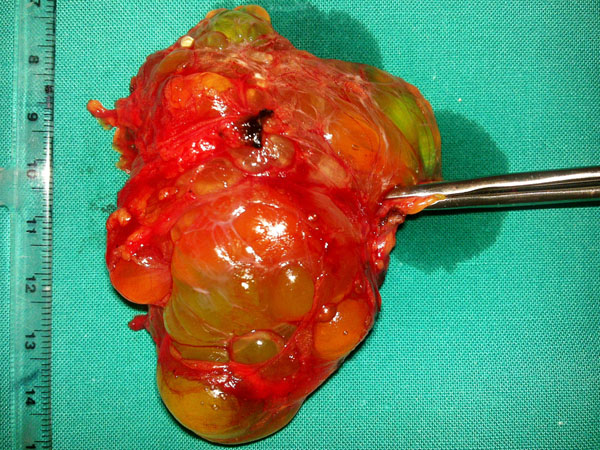
Cystic lymphangioma with polycyclic edge and internal septa (intraoperative specimen)

The patient underwent a laparotomic exploration and the cystic formation was completely excised. (Figure 3)

**Figure 4:**
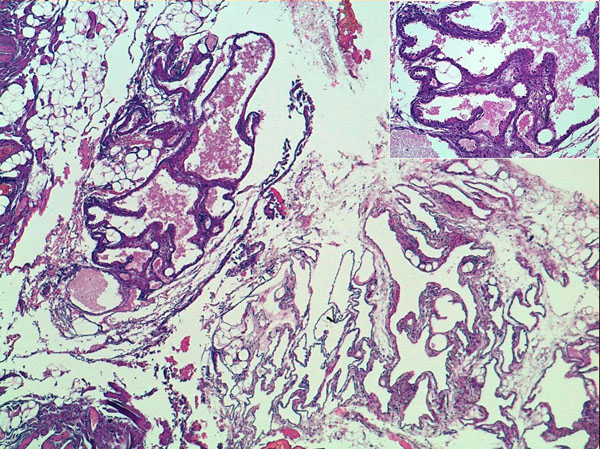
Numerous dilated lymphatic spaces, sometimes filled with lymph (EE 5X). Inset: focally lymphatic spaces are covered by plump endothelial cells (EE 20X)

The histopathological examination was consistent with the diagnosis of mesenteric cystic lymphangioma. (Figure 4)

The patient has been followed-up for 4 months and no recurrence occurred.

## Case report N.4

A 77 – year – old male patient complained of a dull abdominal pain of one year duration localized in the periumbilical region. This pain was associated to abdominal swelling and early satiety after meals.

**Figure 5:**
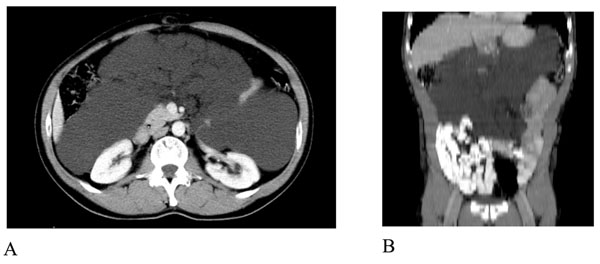
CT of the abdomen. Axial (a) and MPR coronal (b) CT images showed a huge mass composed by multiple confluent cystic lesions occupying the peritoneal spaces and displacing inferiorly the small bowel.

An abdominal examination revealed the presence of a solid mass that occupied the epigastric and mid-epigastric region. An abdomen CT showed a huge mass composed by multiple confluent cystic lesions displacing inferiorly the small bowel. (Figure 5 A - B)

At laparotomic exploration, the mass appeared composed by multiple cysts containing a citrine fluid. A careful dissection was conducted to excise the mass without spillage of fluid in peritoneal cavity.

The histopathological examination confirmed the diagnosis of mesenteric cystic lymphangioma.

The post-operative course showed no complications. The patient has been followed up for 1 year and no recurrence was observed.

## Case report N.5

A 65-year-old male patient was diagnosed ten years before the presence of an intra-abdominal limphangyoma. The mass was followed-up with annual abdominal – MR and ecotomography.

**Figure 6:**
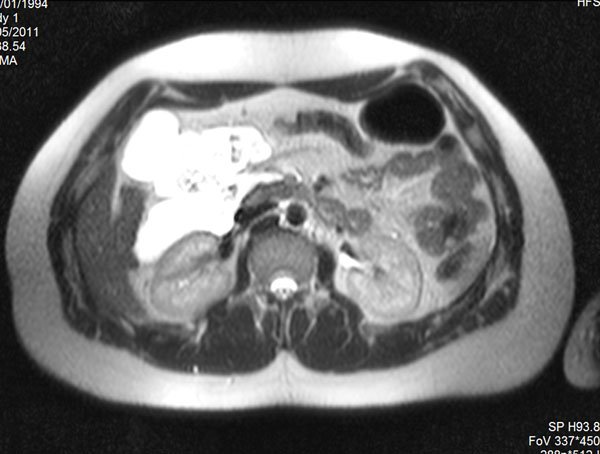
MRI of the abdomen. Fluid - filled mass (18x12 cm) with internal septa located between the stomach and the pancreas with lateral extension in the hepatorenal space.

At abdominal – RM the formation was fluid- filled with internal septa and measured 18x12 cm : it was placed between the posterior aspect of the stomach and the pancreas with lateral extension in the hepatorenal space.(Figure 6) He had no clinical symptoms but due the growing of the mass in the last years (12x8 cm in 2008) we performed a laparotomic resection of the tumor. A citologic examination was conducted on the fluid aspirated from the cyst and it revealed the presence of chronic inflammatory cells. The histopathological examination was consistent with the diagnosis of cystic lymphangioma.

## Discussion

Intra-abdominal mesenteric lymphangioma is an uncommon tumor that is usually manifesting in early adulthood. This tumor has an incidence of approximately 1/20,000 and 1/250,000 hospital admissions, and it is more frequent in men than women with a M/F ratio of 3:1. [4]

Intra-abdominal lymphangioma are almost always found in the retroperitoneum, followed by mesentery. In intraperitoneal sites, the small bowel mesentery (70%) is the commonest site, with 50–60% of all cysts located in the ileal mesentery. [5]

The etiology of mesenteric lymphangioma is considered to be congenital, with abnormal embryonic development of the lymphatic system causing sequestration of lymphatic tissue. However, other possible causes have been suggested such as: abdominal trauma, lymphatic obstruction, inflammatory process, surgery, and/or radiation therapy. [6]

Data relative to our case series are listed in Table 1: we performed the surgical excision of a mesenteric lymphangioma in five patients (4 male and 1 female) with a median age of 71.4 years (65 –80).

**Table 1:** Patient characteristics

	Patient n. 1	Patient n. 2	Patient n. 3	Patient n. 4	Patient n. 5
**Sex**	*F*	*M*	*M*	*M*	*M*
**Age**	*67*	*68*	*80*	*77*	*65*
**Symptoms**	*Abdominal pain*	*Abdominal swelling*	*Intestinal obstruction*	*Abdominal pain and early satiety*	*Asymptomatic*
**Diameter (cm)**	*3,5*	*7*	*8,6*	*15*	*18*
**Congenital abnormalities**	*Multiple pancreatic and renal cysts*	*Left kidney agenesia*	*NO*	*NO*	*NO*
**Histopathology**	*cystic lymphangioma*	*cystic emolymphangioma*	*cystic lymphangioma*	*cystic lymphangioma*	*cystic lymphangioma*
**Follow – up**	*6 months*	*2 years*	*4 months*	*1 year*	*1 year*
**Recurrence**	*No*	*No*	*No*	*No*	*No*

In two patients of our series other congenital abnormalities were associated: in patient n.1 also other cystic lesions (kidney and pancreas) were present and in case report n.2 a left kidney agenesia was associated (Table 1).

In case n.2 the histopatological examination revealed the presence of a cystic emolymphangioma (Table 1) that is very rare entity in intra – abdominal localization. This tumor is considered a congenital malformation of the vascular system whose formation may be explained by obstruction of the venolymphatic communication between dysembrioplastic vascular tissue and the systemic circulation.[7]

In our series, clinical manifestations are variable among patients, with no characteristic signs and symptoms. More common manifestations are a painless abdominal distension or an abdominal mass, with the latter being often detected incidentally. There can be an acute abdomen presentations if the lymphangioma becomes complicated due to infection, hemorrhage or bowel obstruction ( as in case n.3) (Table 1). [8]

The ultrasonographic presentation of a mesenteric lymphangioma is described as a cystic lesion with multiple thin septa (honeycomb or cobweb pattern). Ultrasound-guided diagnostic aspiration usually yields a chylous aspirate. On CT imaging, mesenteric lymphangiomas appear as a uni- or multilocular masses with enhancement of the wall and septum by contrast medium [9]. However these studies help to determine, if the tumor is cystic, its size and location, but they are insufficient to establish an accurate preoperative diagnosis. Magnetic resonance imaging is the most useful preoperative radiological tool for diagnosis and in surgical planning. In differentiating the mesenteric cyst from the lymphangioma, magnetic resonance imaging is suggestive because it allows a good differentiation of cystic and septal structures. Mesenteric cystic lymphangiomas lack demonstrable fat content by chemical shift and fat saturation, as clearly seen in the magnetic resonance imaging of dermoid cyst [10].

However, the definitive diagnosis of lymphangioma is based on histopathology and immunochemistry : the lining mesothelial cells are immunoreactive for cytokeratin and negative for factor VIIIIs. Double staining with Prox1 and CD31 is the most reliable method for characterizing lymphangioma endothelial cells.[11]

In our patients we performed a complete surgical excision of the mass, that is the treatment of choice for cystic lymphangioma, even if asymptomatic. Surgical enucleation without damage to the blood supply of the bowel is also suggested, with the prognosis being generally favorable, but with increasing tumor size radical resection becomes more difficult and local recurrence more probable [12,13]. Infiltration of the intestine or involvement of the main branch of mesenteric arteries or adjacent organs necessitate segmental resection of the intestine [14] .

The prognosis after adequate excision of the cystic tumors of the mesentery is considered to be excellent.

Drainage has been suggested as a modality of treatment in high – risk patients but is often unsuccessful because of recurrence and for the risk of perforation of the mesentery during the drainage of the lymphangioma [15]. Instillation sclerotherapy with alcohol is being used for ablation, but this method can be destructive to normal tisuue. In cases of failed percutaneous sclerotherapy using alcohol, acetic acid has been used with good success in intra-abdominal lymphangioma [16].

Adjuvant treatment with OK-432, a biological response modifier with antitumor effects, has been shown to prevent further enlargement of small localized remnant cysts after surgery [17].

In our experience, we follow up the patients with a clinical examination and an abdominal ultrasound at 3 , 6 and 12 months after the operation , then annually. During a median follow up period of 11,5 months (4 – 48 months) no recurrence occurred (Table 1).

## Conclusion

Mesenteric lymphangioma in adult is a rare disease: it may present as an abdominal swelling or acute intestinal obstruction. Preoperative diagnostic tools are ultrasound and abdomen CT or MR. Complete surgical resection is the ideal modality of treatment for mesenteric lymphangioma. Definitive diagnosis is confirmed by histopathology.
